# Concealing, Connecting, and Confronting: A Reflexive Inquiry into Mental Health and Wellbeing Among Undergraduate Nursing Students

**DOI:** 10.3390/nursrep15090312

**Published:** 2025-08-25

**Authors:** Animesh Ghimire

**Affiliations:** 1 School of Nursing and Midwifery, Faculty of Medicine, Nursing and Health Sciences, Monash University, Wellington Road, Clayton, VIC 3800, Australia; animesh.ghimire@monash.edu; 2 School of Nursing and School of Public Health, Chitwan Medical College, Bharatpur-5, Kailashnagar, Chitwan 44200, Nepal; 3 Health and Education, Sustainable Prosperity Initiative Nepal, Baneshwor-31, Kathmandu 44600, Nepal

**Keywords:** undergraduate nursing students, mental health, reflexive thematic analysis, Nepal, resilience, hierarchy, wellbeing

## Abstract

**Background**: Undergraduate nursing students (UNSs) often enter clinical training just as they are still mastering the emotional labor of the profession. In Nepal, where teaching hierarchies discourage upward dialogue and hospitals routinely struggle with overcrowding, supply shortages, and outward nurse migration, these learners confront a distinct, under-documented burden of psychological distress. **Objective**: This study examines how UNSs interpret, negotiate, and cope with the mental health challenges that arise at the intersection of cultural deference, resource scarcity, and migration-fueled uncertainty. **Methods**: A qualitative design employing reflexive thematic analysis (RTA), guided by the Reflexive Thematic Analysis Reporting Guidelines (RTARG), was used. Fifteen second-, third-, and fourth-year Bachelor of Science in Nursing students at a major urban tertiary institution in Nepal were purposively recruited via on-campus digital flyers and brief in-class announcements that directed students (by QR code) to a secure sign-up form. Participants then completed semi-structured interviews; audio files were transcribed verbatim and iteratively analyzed through an inductive, reflexive coding process to ensure methodological rigor. **Results**: Four themes portray a continuum from silenced struggle to systemic constraint. First, Shrouded Voices, Quiet Connections captures how students confide only in trusted peers, fearing that formal disclosure could be perceived as weakness or incompetence. Second, Performing Resilience: Masking Authentic Struggles describes the institutional narratives of “strong nurses” that drive students to suppress anxiety, adopting scripted positivity to satisfy assessment expectations. Third, Power, Hierarchy, and the Weight of Tradition reveals that strict authority gradients inhibit questions in classrooms and clinical placements, leaving stress unvoiced and unaddressed. Finally, Overshadowed by Systemic Realities shows how chronic understaffing, equipment shortages, and patient poverty compel students to prioritize patients’ hardships, normalizing self-neglect. **Conclusions**: Psychological distress among Nepalese UNSs is not an individual failing but a product of structural silence and resource poverty. Educators and policymakers must move beyond resilience-only rhetoric toward concrete reforms that dismantle punitive hierarchies, create confidential support avenues, and embed collaborative pedagogy. Institutional accountability—through regulated workloads, faculty-endorsed wellbeing forums, and systematic mentoring—can shift mental health care from a private struggle to a shared professional responsibility. Multi-site studies across low- and middle-income countries are now essential for testing such system-level interventions and building a globally resilient, compassionate nursing workforce.

## 1. Background

Mental health and wellbeing are increasingly recognized as critical facets of global health; however, they remain unevenly addressed across various educational and professional sectors. Among these domains, the mental health status of students entering healthcare professions has attracted particular concern, as scholars and policymakers seek to ensure that the future workforce is not only clinically proficient but also psychologically resilient [1,2]. Undergraduate nursing students (UNSs) occupy a uniquely complex position in this discussion. Their academic trajectories are punctuated by an intense blend of theoretical coursework [3], emotionally charged clinical placements [4], and continuous demands on cognitive and affective capacities [5]—a combination that can strain even the most prepared learners. Despite such complexities, the prevalence and nature of mental health challenges in UNSs remain insufficiently explored, especially in low- and middle-income countries (LMICs).

Poor mental health and wellbeing can negatively impact UNSs. Academically, students often experience decreased concentration, diminished decision-making capacities, and reduced motivation for coursework [6]. Psychosocially, anxiety and burnout can accumulate, producing persistent emotional strain [7]. Over time, these stressors can calcify into long-term professional dispositions that impair patient care once the students transition into registered practice. There is a concern that if these adversities remain unresolved during formative training, individuals enter the healthcare workforce more prone to clinical errors, lower job satisfaction, and higher turnover intentions [8]. Such outcomes disrupt personal career trajectories and further burden healthcare systems—particularly in contexts already grappling with acute workforce shortages [9].

### 1.1. Understanding Nepal’s Context

Nepal illustrates many of these challenges in a striking manner. Situated in South Asia and classified as a lower-middle-income country, it faces considerable healthcare system strain due to limited infrastructural resources and the uneven urban–rural distribution of medical personnel [10,11,12]. Nepal is experiencing one of the highest rates of nurse emigration in its region [13,14]. Thousands of Nepali nurses seek employment abroad annually, primarily in such countries as the United Kingdom (UK), Australia, the United States (US), and Canada [15,16]. Difficult working conditions, including high patient loads and resource scarcity, are key factors driving this outward mobility—supported by extensive international recruitment networks that target health professionals and bilateral agreements between the high-income countries (HICs) and Nepal that facilitate skilled migration [17,18]. Culturally, migration is often viewed as a path toward economic and social advancement; however, this ongoing “brain drain” undermines the national healthcare sector, intensifying domestic staffing shortages and limiting the capacity to provide robust mentorship and support for nursing students within local hospitals [10,18].

Although extensive scholarship addresses the professional challenges faced by nurses in Nepal, relatively little research has examined the academic and clinical realities confronting undergraduate nursing students. During these formative years, students transition from predominantly theoretical instruction to immersive clinical practice, internalizing professional codes and ethical frameworks that shape their future delivery of care [10]. Cultural expectations of hierarchical deference further intensify stress, as students often hesitate to voice concerns in rigidly structured environments [18]. Moreover, limited material resources—ranging from overcrowded wards to insufficient medical supplies—magnify the pressures tied to academic workloads [10,19]. As a result, mental health issues among UNSs frequently remain unrecognized or are dismissed as an intrinsic component of nursing education.

### 1.2. Emerging Challenges and Knowledge Gaps

Although research in HICs has identified a correlation between academic loads, clinical demands, and mental health outcomes in nursing students [6,8,20], these findings may not fully capture the unique intersections of emigration patterns, cultural hierarchies, and scarce health resources that characterize LMICs, such as Nepal. Existing studies on Nepalese nursing, moreover, frequently center on registered professionals rather than students [21], overlooking the stage where socialization and coping strategies begin to form. Understanding how stress and psychological distress manifest specifically in UNSs is, therefore, a necessary precursor to developing targeted interventions, both to safeguard trainee wellbeing and to enhance the stability of Nepal’s broader healthcare system.

In the realm of the international nursing literature, resilience, peer support, and the establishment of safe learning environments frequently emerge as recommended strategies for alleviating student stress [22,23]. However, these concepts often presuppose a certain level of institutional resources and cultural openness—conditions that may not be consistently available in LMICs. For example, peer support may be underutilized in settings where vulnerability is discouraged, while interventions focused on resilience may devolve into mere performances if chronic systemic issues, such as understaffing, remain neglected. Given these complexities, a qualitative approach is especially valuable for capturing how individuals navigate and respond to institutional and cultural constraints, thereby providing more nuanced insights into the effectiveness and relevance of proposed stress-mitigation strategies.

### 1.3. Positioning the Present Inquiry

Against this backdrop, a focused appraisal of undergraduate nursing students’ mental health in Nepal becomes essential. Recent national reforms—ranging from curriculum restructuring to faculty-development workshops and expanded clinical placements—have sought to raise the technical caliber of nursing education, yet their psychological consequences remain largely unexamined [10]. By centering inquiry on students rather than on practicing nurses, this study captures a developmental moment when professional values, coping repertoires, and ethical judgements are still forming. Investigating how resource scarcity, hierarchical teaching styles, and migration-fueled career expectations are absorbed—or resisted—during this formative phase offers a critical vantage point for designing interventions that interrupt distress before it calcifies into workplace burnout.

Illuminating the mental health realities of Nepali UNSs also enriches global comparative scholarship. While examinations of nursing education stress in HICs highlight workload, evaluation, financial pressure, and simulation anxiety as near-universal stressors [24,25,26], LMIC contexts add the compounded burdens of infrastructural shortfalls and outward labor flows. Situating the Nepali experience within that broader dialogue clarifies which challenges are ubiquitous to the profession and which are magnified by structural inequity. Moreover, shedding light on UNSs’ mental health in Nepal contributes to a broader comparative dialogue: the findings can contextualize the universal aspects of nursing education stressors, as well as highlight the distinctive challenges that arise in LMICs experiencing high outmigration. Ultimately, such knowledge proves pivotal in both refining local nursing curricula and informing international frameworks that seek to standardize the educational experiences of future nurses [27]. By elucidating the intersection of sociocultural factors, resource constraints, and academic demands, this research underscores the need for a more nuanced, context-specific understanding of mental health in nursing education—one that extends beyond short-term stress management and addresses the systemic drivers that perpetuate distress.

## 2. Theoretical Orientation and Research Aim

### 2.1. Analytic Approach and Theoretical Framework

I employed reflexive thematic analysis (RTA) for this study, guided by the Reflexive Thematic Analysis Reporting Guidelines (RTARG) [28,29]. RTA aligns with my interpretivist and constructionist stance, which posits that social reality and knowledge are co-created through interaction and meaning-making, rather than existing as fixed entities awaiting discovery [30]. This philosophical orientation is especially pertinent to the investigation of mental health and wellbeing among UNSs, whose lived experiences are deeply shaped by cultural, institutional, and interpersonal factors [18].

Within RTA, researcher subjectivity is viewed not as a bias to be eliminated but as an analytic tool that deepens understanding [30]. I intentionally embraced my interpretive role, acknowledging that themes do not simply emerge from the data but are actively generated through my iterative engagement with participant narratives. By adhering to a constructionist perspective, I recognized that the accounts students shared are context-dependent: their descriptions of stress, institutional norms, and aspirations reflect both localized cultural expectations in Nepal and broader discourses on nursing education. This orientation resonates with the reflexive aspect of thematic analysis, wherein my evolving insights—rather than a universal “truth”—inform the analytic process [28].

RTA’s suitability for this inquiry lies in its capacity to unravel how societal structures and personal meaning-making converge in day-to-day student life. In a resource-limited environment, such as Nepal, broader issues (e.g., high nurse emigration, cultural norms of hierarchy) necessitate a form of qualitative inquiry that can illuminate the nuanced interplay of structural pressures and individual responses. Because RTA facilitates both semantic (surface-level) and latent (underlying) analyses [28], I engaged in a balanced interpretive approach—predominantly inductive when exploring the students’ unanticipated concerns, and partly deductive when leveraging existing scholarship on mental health and nursing education. This combination allowed for a robust examination of how widely cited phenomena, such as resilience [22] or institutional support [31], manifest in a setting marked by unique social, cultural, and economic realities.

### 2.2. Researcher Positioning and Reflexive Practice

Reflexivity is central to RTA and formed an integral dimension of my analytic process. Over eight years of immersion in Nepal’s healthcare field—initially as a clinical nurse, then as a nurse educator and a policy-oriented researcher—I have observed incremental shifts in the attention afforded to student mental health within nursing curricula, alongside persistent structural strains. Earlier iterations of training prioritized clinical competence with scant acknowledgement of wellbeing; more recent efforts to incorporate self-care and peer-support content remain uneven and resource-constrained. My dual experience across remote, rural posts and high-volume urban centers sensitized me to how short staffing, inconsistent supplies, and cultural deference to authority shape students’ emotional lives. At the same time, I recognized that my insider status could predispose me to normalize stoicism or under-read distress. Accordingly, I employed a set of reflexive strategies designed to surface and check my assumptions throughout the study.

First, I kept a structured reflexive journal after every interview (methodological notes, positionality memos, and analytic hunches recorded with date/time stamps). These entries documented when my educator perspective might be coloring interpretations and specified remedial steps (e.g., seeking counter-examples before fixing a code). A brief, de-identified excerpt illustrates this practice, as follows:

Reflexive journal, 18 February 2025: “I notice myself nodding when P6 frames ‘being strong’ as professional pride. That is my educator reflex. Before coding this as ‘competence’, I need to ask: what were the costs she did not voice? Add a prompt in the next interview to probe ‘what happens after you act strong?’ and look for extracts that complicate the ‘strength = success’ link”.

Second, I scheduled peer debriefings with two colleagues external to the study (a qualitative methodologist and an allied-health educator) at three points (after the 5th, 10th, and final interview). These sessions functioned as “critical-friend” dialogues: they interrogated my coding rationales, pressed for alternative readings, and asked whether labels (e.g., “resilience”) risked importing normative judgments. Action items from each debriefing were logged (e.g., retain separate codes for “quiet peer solidarity” and “individual faith practices”; test disconfirming evidence for the emerging “self-silencing” construct).

Third, I used bracketing routines before analysis blocks and interviews (“stop–question–reframe”). I explicitly wrote down anticipated narratives (e.g., “students value composure”) and then posed counters (“what if composure is a protective mask rather than an endorsed ideal?”). During coding, any passage that fit my expectations “too neatly” triggered a red-flag review: I sought discrepant excerpts and revisited label wording to preserve interpretive openness.

Fourth, I maintained an audit trail/decision log (versioned code lists, theme name changes, and justifications). When renaming a provisional theme from “Culture of Silence” to “Shrouded Voices, Quiet Connections”, the log notes record why private peer exchanges (not silence per se) were central to participants’ meaning-making and how this reframing altered inclusion criteria for extracts.

Finally, I practiced in situ member reflections during interviews (brief, clarifying paraphrases and invitations to nuance—e.g., “Have I got that right, or is there more to how you experienced this?”). Consistent with RTA, I did not conduct post hoc member checking of themes; rather, these real-time reflections supported situated accuracy without implying a single authoritative reading.

Taken together, journaling, critical-friend debriefings, bracketing, a documented audit trail, and in situ reflections helped sustain a productive tension between my insider knowledge and the analytic distance required for reflexive interpretation. These practices were intended to make presuppositions visible, invite alternative readings, and preserve multiple possibilities before settling on the most coherent thematic story.

### 2.3. Research Aim and Questions

Building on this theoretical and reflexive grounding, I sought to generate an in-depth understanding of the psychological and emotional challenges faced by UNSs within Nepal’s distinct sociocultural and institutional context. In this research, psychological distress is conceptualized broadly to encompass manifestations of anxiety, burnout, and emotional strain related to academic workloads, clinical exposures, and broader systemic factors, such as limited resources and cultural norms of hierarchy. Recognizing the multifaceted nature of mental health, I formulated the following two research questions to capture the complexity of these experiences:

How do undergraduate nursing students in an urban tertiary institution in Nepal perceive, navigate, and cope with psychological distress during their clinical training?

In what ways do cultural norms, hierarchical structures, and resource constraints shape the expression, management, and potential resolution of these mental health challenges among undergraduate nursing students?

By framing the inquiry through these two questions, I aim to illuminate not only the lived realities of students’ mental health challenges but also how broader environmental and cultural factors uniquely influence the ways in which psychological distress is recognized, articulated, and potentially addressed.

## 3. Methodology

### 3.1. Study Setting, Participant Selection, and Characteristics

#### 3.1.1. Study Setting

Chitwan Medical College is located in Bharatpur, an urban center within Bagmati Province, Nepal [32,33]. It offers comprehensive healthcare services and advanced nursing programs as a tertiary-level teaching institution [33]. Although the region benefits from a relatively developed health infrastructure compared to rural counterparts [32], resource constraints still pose substantial challenges, including limited staffing, high patient demand, and significant financial barriers for local residents seeking care. These realities create a rich environment for exploring how UNSs experience and manage psychological distress. Because the college admits one of Nepal’s largest cohorts of nursing students, it provides an ideal site to study how future healthcare professionals learn, adapt, and cope in a resource-limited yet academically demanding context.

#### 3.1.2. Participant Selection and Information Power

I used a purposive selection strategy [34] guided by information power rather than statistical representativeness [35]. This decision aligned with the study’s interpretive aim to generate depth and nuance about psychological distress. Eligible participants were (1) second-, third-, or fourth-year Bachelor of Science in Nursing students and (2) actively engaged in clinical rotations. First-year students were intentionally excluded because they have limited clinical exposure and are still acclimating to university demands, making them less able to speak to the study’s focus on clinically linked forms of distress.

At Chitwan Medical College, undergraduate nursing students undertake clinical placements in both urban tertiary hospitals and rural/primary care sites. Because the institution is based in an urban center and the majority of clinical hours during the data collection period were scheduled within affiliated urban tertiary hospitals, the research question was framed to foreground the urban tertiary context. This choice was purposive (i.e., theoretically and contextually coherent) and also pragmatic in terms of feasibility and access to students at the study site.

Rather than setting demographic quotas, I sought participants prepared to offer rich, reflexive accounts of mental health and coping in relation to academic workload, clinical demands, hierarchical norms, and material constraints. Recruitment materials described this focus so that volunteers self-selected on the basis of experiential relevance. Fifteen students—four second-year, five third-year, and six fourth-year—took part. In line with information-power reasoning, this sample was judged adequate given the narrow aim, relatively specific participant group, strong quality of interview dialogue, and good coverage across the three senior cohorts [35]. Consistent with reflexive thematic analysis, the goal was not “saturation” in a positivist sense but sufficient information power to support credible, situated interpretation.

#### 3.1.3. Recruitment and Selection Process

Recruitment for this study spanned four months from December 2024 to March 2025. To ensure voluntary participation and mitigate perceived institutional pressure, digital flyers were displayed in student common areas and academic corridors. Each flyer contained a quick-response (QR) code that directed interested individuals to a secure online form, where they could read detailed information about the study’s purpose, data confidentiality measures, and their right to withdraw at any stage.

No incentives were offered, and students were explicitly informed that their decision to participate—or not—would not influence their academic evaluations. Once prospective participants completed the online form, they were contacted via email for a brief confirmation and scheduling of a convenient time to discuss consent. Those who confirmed their willingness to participate provided verbal and written consent before further engagement with the study. As part of this process, students received assurances of anonymity and confidentiality, including how their personal information would be de-identified in all published materials.

In total, 15 UNSs volunteered. This distribution reflected the purposive selection rationale of capturing diverse academic stages and experiences with clinical rotations. The four-month timeline allowed sufficient opportunity for interested students to review study details, consult with peers or faculty if desired, and register without feeling rushed. This measured approach fostered a balance between inclusivity—allowing different class schedules and commitments—and maintaining a clearly defined recruitment window that aligned with the broader objectives of the research.

#### 3.1.4. Reflexive Considerations in Recruitment

I occupy dual roles as a nurse educator and a researcher with policy engagement. While my institutional familiarity facilitates rapport with nursing students, it can also introduce implicit power dynamics. To mitigate potential coercion or self-censorship, I deployed private sign-up procedures via the QR code and reiterated the independence of this study from academic grading. I also kept a self-reflexive diary, noting any assumptions or biases I brought to each interaction—particularly in relation to students’ perceived academic performance or alignment with established nursing norms. These steps aimed to foster a climate where participants felt secure disclosing intimate thoughts on stress, anxiety, and peer/faculty relationships.

#### 3.1.5. Participant Characteristics

All 15 participants were female (100%), reflecting broader demographic trends within Nepalese nursing where the workforce is entirely female [36]. Ages ranged from 23 to 28 years (median = 25; interquartile range (IQR) = 24–26). Table 1 provides the individual-level summary by year of study and first-choice career status; in aggregate, 4 of 15 participants (26.7%) were in the second year, 5 of 15 (33.3%) were in the third year, and 6 of 15 (40.0%) were in the fourth (final) year. Ten of the fifteen participants (66.7%) reported nursing as their first-choice career, indicating strong intrinsic motivation to pursue nursing at the undergraduate level. Typically, Nepalese students enter the BSc Nursing track after completing high school (Year 12), embarking on a four-year curriculum that integrates theoretical instruction with progressively more complex clinical experiences [37]. The second-year cohort had gained initial exposure to hospital wards, the third-year group had assumed more extensive responsibilities, and final-year students were nearing completion of their qualifications. Some participants aspired to international migration after graduation, while others intended to remain in Nepal, underscoring heterogeneous longer-term trajectories within a uniformly female cohort.

### 3.2. Ethical Considerations

This study was approved by the Nepal Health Research Council (NHRC-245/2024) and the Institutional Review Board of Chitwan Medical College in November 2024. All participants were thoroughly briefed on the nature, purpose, and scope of the research through written and verbal explanations. The informed consent procedure underscored the voluntary nature of participation, clarifying that students could decline or withdraw at any time without academic or personal repercussions. Moreover, the consent form was distributed and signed by all 15 participants. Recognizing the potential for perceived power imbalances in a university setting, it is noteworthy that the researcher did not directly instruct any participating students and had no prior relationship with them. This arrangement further minimized the risk of undue influence and ensured that participants felt secure and autonomous in their decision to contribute to the study. To safeguard participant wellbeing, a brief distress-management protocol accompanied each interview. Before commencement, students received an information sheet listing on-campus counselling and student-wellness services available free of charge at the institution, as well as national helpline details. Interviews could be paused or discontinued at any point, and each session concluded with a short check-in to assess comfort.

### 3.3. Dataset Generation: Method, Tool, Procedure, and Preparation

#### 3.3.1. Method

A reflexive thematic analysis (RTA) design provided the foundational structure for examining how UNSs in Nepal perceive and manage psychological distress. In alignment with the Reflexive Thematic Analysis Reporting Guidelines (RTARG) [28], one-on-one semi-structured interviews served as the primary method of data generation. To enrich participant recall and facilitate deeper reflection, students were encouraged—though not mandated—to keep personal reflective diaries before their scheduled interviews. These brief notes focused on daily emotional states, notable stressors, and informal coping strategies. No diaries were physically or electronically collected; rather, participants were free to refer to their notes during the interview as a memory aid. This arrangement upheld participants’ privacy and simultaneously leveraged the benefits of real-time self-reflection to elicit richer narrative accounts.

#### 3.3.2. Tool Development

The semi-structured interview guide was shaped by a literature review of mental health studies in nursing education and insights from my experiences as a nurse educator in Nepal. This enabled the questions to reflect known stressors, such as resource limitations, migration pressures, and hierarchical norms. To ensure methodological rigor consistent with RTA, I consulted a “critical friend” [30], an experienced qualitative researcher who provided constructive critique of my questioning framework. Within reflexive thematic analysis, a critical friend fosters enhanced reflexivity by challenging implicit assumptions or cultural biases, thereby promoting transparency over any notion of objective “accuracy”. After multiple rounds of feedback, the guide was refined to encourage participants to discuss academic workloads, stress triggers, cultural expectations, and coping responses in depth (Table 2).

#### 3.3.3. Procedure

All interviews were conducted in a private, quiet room at Chitwan Medical College, with each session lasting up to 60 min. Although English is the principal medium of instruction in Nepal’s nursing education—indicating that students are fluent—participants could respond in whichever language (English or Nepali) felt more natural. This flexibility aimed to reduce linguistic barriers and allow for more authentic disclosure of personal experiences.

#### 3.3.4. Data Capture and Preparation

Each interview was audio-recorded with the participant’s written and verbal consent, then transcribed verbatim. Any references participants made to their diaries were captured in the interview transcripts but remained anonymous. Identifiable information was removed, and each transcript was assigned a code (e.g., P1, P2) to protect confidentiality. Following reflexive thematic analysis, these interview transcripts formed the central dataset for subsequent interpretive work. Through this approach—combining semi-structured interviews with prior individual reflection—both researcher and participants could explore situational, emotional, and cultural facets of psychological distress consistent with RTA’s emphasis on depth and context-specific meaning-making.

### 3.4. Data Analysis: Analytic Process and Theme Construction

After completing and reviewing all transcripts, the analytic process for this study followed RTA principles, moving through iterative cycles of coding and theme development. Below is a detailed account of how I engaged with the data, leading to the final set of themes.

#### 3.4.1. Familiarization and Initial Coding

The analysis began with manual transcription of each audio-recorded interview. This transcription readied the data for coding and served as a foundational “familiarization” phase, during which I annotated significant expressions, recurring stressors, and potential contextual factors (e.g., peer relationships, resource constraints). I then performed a first pass of interpretive coding, marking segments of text that captured participants’ experiences of psychological distress and coping. These initial codes were intentionally broad—such as “hidden anxiety”, “family pressure”, or “financial guilt”—to avoid prematurely foreclosing on interpretive possibilities.

All coding and theme development were conducted by the sole analyst. Within reflexive thematic analysis, a single-analyst approach is methodologically coherent because analysis is explicitly interpretive and led by the researcher, rather than a procedure seeking intercoder agreement [28,30]. To make the influence of my standpoint transparent and to mitigate the risks of solitary analysis, I put several safeguards in place, as follows: I kept a dated decision log that linked raw quotations to evolving meaning units and candidate themes; I wrote iterative analytic memos to record hunches, uncertainties, and rival readings; I repeatedly returned to full transcripts to seek disconfirming evidence and test the fit of codes; and I engaged a critical friend for sense-checking of emergent interpretations (the critical friend did not code data or adjudicate themes). These practices were intended to surface and scrutinize my assumptions, preserve a clear audit trail of analytic moves, and retain an interpretive, reflexive stance consistent with RTA while minimizing over-reliance on any single reading of the data. I acknowledge that my background as a Nepal-based nurse educator may have sensitized me to hierarchy and resource constraints; the reflexive procedures above were used deliberately to prevent overemphasis on these issues where not supported by participants’ accounts.

#### 3.4.2. Refining Codes and Iterative Clustering

A second cycle of coding involved revisiting each transcript with the initial codes in mind. This time, I grouped codes that appeared to express overlapping ideas. For instance, “fear of judgment from faculty” and “reluctance to complain about exhaustion” converged around the concept of “self-silencing”, prompting me to collapse those codes into a single category. Conversely, some segments diverged in unexpected ways. A few participants framed “peer support” as vital for dealing with institutional stress, while others described relying primarily on personal faith. These differences led me to keep “peer connections” and “individual coping mechanisms” as separate codes to honor the varied perspectives.

Throughout this phase, I engaged in analytic memoing, noting my evolving interpretations and reflecting on my own potential biases—particularly my familiarity with common tropes in nursing education. This reflexive practice allowed me to remain open to new or contradictory data, rather than forcing them into preconceived categories.

#### 3.4.3. Theme Construction and Labeling

Following the iterative clustering, I generated provisional themes by focusing on the central organizing concepts that united multiple codes. For instance, codes related to hidden anxieties, quiet peer discussions, and perceived stigma about admitting weakness coalesced into an emerging theme. Initially, I labeled this set of ideas as “Culture of Silence”. However, a closer look revealed that many participants also emphasized covert peer relationships as a lifeline, rather than pure silence. Consequently, I re-labeled the theme “Shrouded Voices, Quiet Connections” to capture both the concealment of emotional distress and the discreet peer networks formed to address it.

As I examined how themes converged or diverged, I fine-tuned the overarching categories. For example, one participant’s references to performing resilience around senior staff aligned with another participant’s remarks on “keeping up appearances”. Initially, these appeared under different codes—“masking emotions” and “impression management”—but the similarity in how participants described presenting a “strong front” suggested merging them under a unified concept, namely “Performing Resilience: Masking Authentic Struggles”.

#### 3.4.4. Review and Definition of Final Themes

To consolidate the analysis, I undertook repeated cycles of reviewing and refining candidate themes against the full dataset, returning to coded segments and analytic memos to check scope, coherence, and distinctiveness. In keeping with Big Q qualitative values and reflexive thematic analysis (RTA), I did not apply small q/positivist procedures (e.g., intercoder agreement, consensus coding, or a numerical “data saturation” threshold). Instead, I treated researcher subjectivity as an analytic resource and relied on continued memoing and decision-logging to guide iterative patterning across the corpus [38,39].

Consistent with these commitments, I worked to an interpretive endpoint of thematic saturation rather than “data saturation”: that is, a point at which the analysis appeared robust in capturing the central concepts, and further analytic engagement yielded minimal novelty or modification to the architecture of the themes [35]. This position aligns with reflexive TA’s critique of saturation-as-closure and its emphasis on situated, provisional meaning-making; while additional participants might always surface new nuances in a sensitive topic, such as mental health, the analysis was judged sufficiently elaborated for the study’s aims when subsequent cycles refined rather than substantively reconfigured candidate themes [35,40].

On this basis, I finalized the four themes by (a) checking that each theme had a clear central organizing concept; (b) pruning overlap at theme boundaries; and (c) retaining “border” extracts where interpretively warranted, while documenting the rationale in the decision log. This recursive confirmability process favored transparency over pseudo-objective certainty and is congruent with the RTARG’s guidance for situated, reflexive reporting [28,30,41]. Table 3 offers an illustrative example of this progression, tracing how meaning units advanced from initial interpretive segments to final theme labels. In keeping with reflexive transparency, a representative segment of the analytic decision trail is provided in Supplementary File S1 (Table S1), illustrating how selected meaning units were worked with interpretively (via provisional labels, reflexive memos, and conceptual groupings) toward the four reported themes. This table is illustrative rather than exhaustive and reflects an iterative, constructed process consistent with RTA.

## 4. Analysis

**Figure 1:**
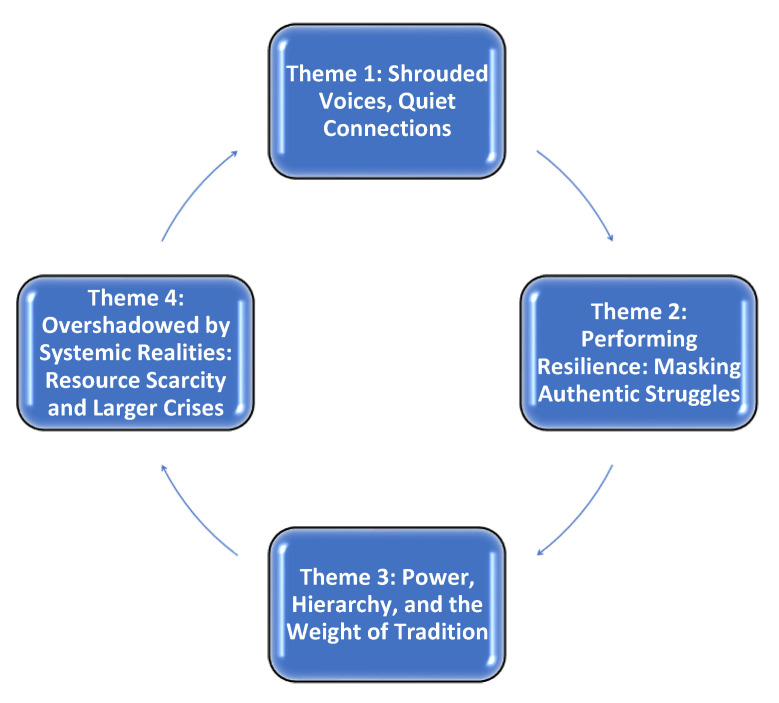
Thematic map depicting the interplay of four emergent themes.

At the heart of the students’ accounts were deeply interwoven experiences that collectively shaped their mental health and wellbeing. These stories highlighted not only the challenges of learning to care for others in a demanding environment but also the societal and institutional expectations that framed—or sometimes curtailed—their ability to seek help. As the analysis progressed, four central patterns emerged, each illuminating a distinct facet of how participants grappled with their emotional and psychological needs within this context. In combination, these themes form a cohesive interpretive tapestry depicting how cultural norms, perceived resilience, hierarchical power structures, and systemic resource constraints converge to affect UNSs’ day-to-day realities. Figure 1 presents a thematic map capturing the dynamic interconnections among these four themes. By situating the findings in a cyclical configuration, the map foregrounds the idea that no single influence operates in isolation; instead, each domain of experience flows into and shapes the others.

### 4.1. Theme 1: Shrouded Voices, Quiet Connections

A pervasive sense of guardedness around personal distress emerged as one of the most striking features of participants’ accounts. While most UNSs acknowledged experiencing moderate to high levels of stress, very few felt able to voice these concerns openly in formal academic or clinical spaces. Instead, students typically relied on a web of informal peer relationships—a network of hushed corridor discussions, late-night text exchanges, and whispered reassurances—that provided a crucial counterbalance to the silence enforced by broader cultural and institutional expectations. This dynamic interplay between self-censorship and peer-based support forms the central organizing concept of “Shrouded Voices, Quiet Connections”.

Several participants noted how peer relationships served as a “safe haven”, especially when they encountered challenging situations in the clinical setting or felt overwhelmed by academic requirements. One student explained succinctly, “We have to keep a lot of things to ourselves. But sometimes it’s just that one friend in class you can text at midnight and say, ‘I can’t take this anymore,’ and they get it.” (P7, third-year student). Such brief moments of informal disclosure appeared to relieve some of the tension caused by an otherwise restrictive environment—an environment in which talking about mental health could be taken as an admission of weakness or a lack of resilience.

Although reliance on peers offered solace, it did not negate the profound constraints on open conversation. Many participants perceived that institutional structures—both at the college and in the broader Nepalese healthcare culture—privileged stoicism, reinforcing a code of emotional reticence similar to the “don’t ask, don’t tell” ethos documented in prior investigations of healthcare education [18,42]. As illustrated by the following quote, the reluctance to speak out was rooted in fear of judgment by both faculty and fellow students:

“Sometimes, after a difficult shift in the ward, all I wanted was to tell someone I felt like I was drowning. But if I tried to mention it in front of seniors or instructors, I’d get responses like, ‘Welcome to nursing—if you can’t handle it now, how will you manage in real practice?’”

“It made me feel inadequate, so I just kept it in. I realized the only safe place to share was with a small circle of classmates who would say, ‘Me too,’ or ‘You’re not alone.’”

(P13, fourth-year student)

These findings align with discussions in the literature that frame nursing education as an inherently high-pressure pathway where unspoken norms often pressure students to conceal vulnerability [43]. According to the umbrella review [44], such concealment is particularly heightened in contexts where professional expectations valorize fortitude, potentially compounding the risks of burnout and attrition. In the present study, the weight of these implicit expectations was underscored by students’ descriptions of an “always on” culture, making it difficult to voice mental health struggles even when institutional protocols purportedly supported wellbeing initiatives.

At the same time, the covert methods of peer support described here reflect a broader pattern found in international studies of nursing education, where communities of practice develop beyond the formal curriculum [9,45]. While the “official” academic discourse may remain silent on mental health challenges, students themselves create micro-communities of understanding, reminiscent of the support circles noted in the study by Zhou et al. [46]. According to the grounded theory analysis, reflective gatherings were shown to be beneficial in promoting psychological wellbeing among nursing cohorts [47]. Similarly, participants in the current research utilized discreet gatherings—often consisting of just two or three people—for similar purposes, but these were student-driven rather than institution-sponsored.

Not all participants, however, embraced the discreet peer model. Several feared that confiding even in friends might spark rumors or jeopardize their reputations. One participant offered the following contrasting viewpoint:

“I don’t share anything with classmates because, in the end, I’m afraid it’ll become gossip, or someone will see me as weak. I’ve noticed that the same friend who offers comfort one day might joke about it with others the next. So, I’d rather just keep my feelings hidden.”

(P3, second-year student)

This divergence implies that while peer connections alleviate stress for many, they can simultaneously pose a risk to confidentiality. Hence, the notion of “quiet connections” is not universally perceived as stable or fully dependable. These complicated dynamics highlight the precarious balance between community-based coping strategies and interpersonal trust—particularly salient in cultures that emphasize collective harmony, sometimes at the expense of individual disclosure [48].

Situated within the existing literature, the accounts here introduce two vital contributions to scholarly dialogues on nursing student wellbeing. First, they reveal the significance of *informal, student-led safety nets* in the absence of robust institutional frameworks. Earlier works have pointed to peer mentoring programs as a structured intervention [49], but the present study underscores the organic ways in which students adapt and craft shadow networks of support, away from official oversight. Second, these findings spotlight the *complex interplay* between cultural norms that reward emotional restraint and the increasing push toward resilience and self-care narratives in nursing education [50]. The interplay is particularly vivid in low-resource contexts, like Nepal, where the moral imperative to uphold professional competence frequently eclipses discussions of personal wellbeing [18].

### 4.2. Theme 2: Performing Resilience: Masking Authentic Struggles

A recurring motif in participants’ narratives was the tension between cultivating genuine resilience and merely appearing to do so in order to meet external expectations—a phenomenon aptly termed “performing resilience”. While officially promoted as a desirable attribute in nursing school, resilience became, for many students, a kind of performance in which emotional labor was quietly obscured behind a veneer of composure. Participants described how institutional messaging around self-reliance, combined with a broader societal ethos that discourages “complaining”, pushed them to appear calm and collected despite significant psychological strain. This performance of resilience reflects the broader cultural tendency in Nepal to equate emotional expression with vulnerability, as has also been documented in other South Asian healthcare contexts [51,52].

One participant, reflecting on her early clinical rotations, offered the following succinct observation: “It feels like you have to be unbreakable, even when everything in you is telling you to stop” (P4, second-year student). The emphasis on unwavering fortitude was reinforced not only by senior faculty but also by peers who had internalized the same “toughness” paradigm. Yet this expectation often ran counter to the lived experience of exhaustion, sleeplessness, and emotional distress. Several students described frequent episodes of anxiety or low mood but hesitated to reveal these states openly, as follows:

“In my first year, I cried almost every night—over exams, ward duties, everything […]. But I never let anyone see it. If someone asked me, ‘How are you holding up?’ my standard reply was ‘I’m good, just a bit busy.’ We get told that a ‘real nurse’ handles pressure without fuss, so it felt like I had no choice but to pretend.”

(P12, fourth-year student)

This tendency to deny or minimize personal distress is reminiscent of what Efstathiou et al. [44] describe as the “anxiety–silence interplay”, where overemphasis on stoicism leads students to internalize—and hide—their emotional needs. Yet this study extends those observations by showing how expressions of resilience can be used to mask, rather than confront, underlying struggles. Moreover, many participants here felt that “resilience talk” from instructors or administrators often had the unintended effect of shaming those who could not immediately conform to the standard of personal endurance.

A key contribution of these findings relates to the concept of “deferred self-care”, where participants deliberately postponed attending to their emotional or psychological concerns until they met external markers of competence, such as passing clinical assessments. Instead of seeking timely professional help or institutional support, students placed their mental health “on hold”, fearing that open admission of vulnerability might jeopardize their perceived suitability for the nursing profession. This practice of deferred self-care not only increases the risk of burnout but also undermines genuine resilience-building, which relies on acknowledging personal limits and leveraging external resources in a timely manner [53].

One student recounted an occasion when she almost fainted in the ward due to severe fatigue but insisted on carrying on, as follows:

“I could barely stand straight. My head was spinning, and I knew it was because I hadn’t slept properly for days. But there was this voice in my head saying, ‘If you stop now, everyone will think you’re not strong enough for nursing.’ So, I kept going—finished the shift, went home, slept maybe three hours, and came back the next day.

It’s not that I didn’t want to rest—I desperately needed it—but I didn’t want to be seen as weak or incapable, especially in front of other nurses and students.”

(P4, second-year student)

Such experiences underscore how the veneer of resilience can foster a potentially hazardous cycle, where students push themselves beyond safe limits to maintain appearances. These findings resonate with a broader debate on the ethics of emphasizing resilience without sufficient systemic support [54]. Scholars argue that while cultivating personal resilience is beneficial, it risks placing undue responsibility on individuals if the broader work or study environment remains unresponsive to their actual needs [55].

The concept of “performative resilience” also intersects with gender norms in nursing, a traditionally female-dominated profession in Nepal. Nursing students often face additional societal expectations to uphold familial and community roles, amplifying the pressure to exhibit unflagging emotional equilibrium [18]. Although some participants alluded to social or familial duties as further stressors, only a minority explicitly connected these to how they outwardly performed resilience. This nuance suggests that while certain macro-level cultural codes influence students’ behavior, individual interpretations of these codes vary considerably.

Nevertheless, it is vital to note the diversity in how students responded to perceived pressures to “act strong”. A few participants identified specific coping skills—like journaling, yoga, or short mental health breaks—that allowed them to manage distress more constructively. Several also found that small-group settings—akin to the “quiet connections” from Theme 1—reduced the need to perform resilience. These pockets of authenticity offered moral support and a chance for more open expression, reinforcing the notion that resilience, when nurtured through collective understanding, can be authentic rather than staged.

### 4.3. Theme 3: Power, Hierarchy, and the Weight of Tradition

A pervasive culture of deference to authority emerged as another key factor influencing mental health among undergraduate nursing students. In Nepal’s tightly woven social structures, respect for seniority and institutionalized hierarchies often shapes interpersonal exchanges—a pattern that intensifies within medical and nursing education [18,56]. Participants recounted how navigating these layers of hierarchy could foster uncertainty and apprehension, particularly when seeking support or voicing concerns.

Many students described scenarios in which they felt compelled to accept responsibilities or stressful workloads beyond their capacity. One particular observation captured the situation as follows: “Even if I’m overwhelmed, I’d rather not say no to a senior lecturer or preceptor. In our tradition, you don’t question what your elders or supervisors tell you to do” (P15, fourth-year student). This tendency to comply, even when personal wellbeing might be at stake, reflects a broader sociocultural inclination to avoid “disrespect” by challenging higher authorities [18]. While hierarchy can foster mentorship structures in some contexts [57], it often stifles open dialogue about mental health and wellbeing in this study.

Beneath the surface, students felt the pull of long-held cultural expectations that a conscientious nurse is someone who acts with humility and deference—attributes that further complicate attempts to assert emotional or psychological needs [58]. Unlike some Western contexts where questioning superiors is considered evidence of independent thinking [59], participants here worried that raising issues, such as burnout or anxiety, might be misconstrued as insubordination. One student summed up the dilemma as follows:

“We keep hearing that we should be assertive if we feel unsafe or if the workload is too much. But the moment we try to speak up, it can be seen as a challenge to authority. I’ve actually been told, ‘Know your place. You’re just a student; it’s your job to adapt.’ That makes it really difficult to ask for help.”

(P8, third-year student)

These findings align with broader research showing how cultural and institutional hierarchies can significantly shape the nursing student experience [60]. However, the present study adds the nuance that such hierarchical norms not only discourage open communication about clinical or ethical dilemmas—issues documented in earlier studies of nursing education [18]—but also further suppress open expression of personal mental health struggles. Hierarchical pressures in nursing education are frequently disguised under the rubric of “professional socialization”, thereby framing reticence to question authority as an inherent part of nurse training [18].

A notable dimension of this theme was how gender norms intertwined with hierarchical power. Nursing, often conceptualized in Nepal as a “female profession”, [36] carries an undercurrent of traditional roles, which can intensify pressures to exhibit unqualified compliance [10]. P5, a third-year student, observed that, “Being a woman in a patriarchal setting is already restrictive, and being a nursing student in a strict hierarchy doubles it.” While not all participants attributed their mental health challenges explicitly to gendered power imbalances, such reflections highlight how multiple axes of tradition—such as patriarchal norms and professional hierarchies—can compound the pressure to remain silent.

Despite the weight of these ingrained customs, students were not entirely passive. Several described small acts of resistance, such as discretely sharing feedback among peers before cautiously approaching faculty. Others formed alliances with more sympathetic senior staff who were willing to mediate between students and rigid institutional protocols. However, these actions often remained informal and covert, illustrating a phenomenon akin to “quiet negotiation”. According to Mailyan [45], quiet negotiation in hierarchical educational contexts can serve as an adaptive strategy that allows marginally empowered groups—like UNSs—to obtain minimal concessions without overtly challenging the status quo. The present study provides further evidence of these adaptive behaviors, suggesting that while hierarchy exerts a powerful gravitational force, its effects are neither monolithic nor absolute.

Another consequence of unyielding hierarchy was the perceived erosion of collaborative learning. In contrast to models of “active learning” advocated by many nursing pedagogies in high-income countries [61,62], participants here believed that top-down instructional methods limited critical discussion and mutual support. One student shared the following:

“During ward practice, we are often just expected to follow orders from senior nurses or doctors. If we fail or struggle, it’s on us. I remember trying to mention that I was repeatedly assigned the busiest tasks, and I was feeling overwhelmed. The head nurse told me, ‘This is how you learn resilience.’ There was no real conversation about balancing the workload or providing support. That’s the moment I realized we’re not encouraged to think collectively; it’s everyone for themselves, adhering to the chain of command.”

(P10, fourth-year student)

This dynamic has implications for mental health. While proponents of hierarchical systems sometimes argue that clear chains of command expedite decision making [63], participants in this study rarely found such a structure conducive to their emotional wellbeing. Instead, they repeatedly underscored how it fostered a “look after yourself” mentality that conflicted with any notion of collective resilience or genuine collaboration. The tension echoes what Solomon et al. [9] identified as a “cultural mismatch” between aspirational nursing values—like empathy and mutual support—and the hierarchical norms that overshadow them in practice.

### 4.4. Theme 4: Overshadowed by Systemic Realities: Resource Scarcity and Larger Crises

Participants frequently portrayed their own mental health struggles as secondary to the relentless resource limitations and financial dilemmas they encountered during clinical placements. Many students felt their emotional wellbeing was simply overshadowed by what they perceived as much larger systemic problems, such as frequent shortages of equipment, high patient loads for overstretched ward staff, and families who could barely afford the costs of care. One student reflected on the dissonance she experienced in the hospital environment as follows:

“I remember feeling tired and anxious after days on the ward, but then seeing how some patients had no money to pay for treatments […] and were forced to choose whether to go ahead with the treatment plan or not […] or some patients taking loans to pay their medical bills […]. It made me think, ‘Who am I to complain about my stress when people can’t even pay for their treatment?’ So I just kept my worries quiet.”

(P4, second-year student)

This sentiment echoed a sense of moral responsibility in which students deprioritized their own wellbeing out of empathy for patients’ hardships. While professional norms often emphasize compassion, participants suggested that identifying these broader crises, particularly insufficient resources and patient poverty, led them to downplay personal distress. Their accounts resonate with the notion of moral incongruence, wherein individual psychological needs are viewed as less “worthy” in light of perceived institutional urgency [64]. Such internalized comparisons can perpetuate a cycle of guilt and self-silencing, effectively stalling any candid expression of mental health difficulties.

The magnitude of these systemic challenges also seemed to exert pressure on students to adapt quickly or risk appearing ill-suited for the demands of healthcare work. One of the students highlighted the following:

“Sometimes there aren’t enough medical supplies […], and nurses tell me, ‘We have to make do.’ I understand that’s real life here, but it makes me anxious because I’m still learning and need a certain level of structure. But it feels selfish to say that out loud when everyone else is just ‘dealing with it.’”

(P9, third-year student)

Here, resource shortages intersect with a “make do” ethos that pervades the clinical environment. Students felt they had little choice but to follow along, reluctant to raise concerns for fear of seeming fragile or unprofessional. While some degree of adaptability is integral to nursing education, the intensity and frequency of these shortages created a culture where coping was elevated to a near-heroic ideal. This valorization of constant crisis management inadvertently sidelines any meaningful conversation about student burnout or anxiety. Moreover, it extends the findings from earlier themes by revealing how broader structural constraints—such as underfunding and minimal staffing—reinforce the reluctance to seek help, even when mental health resources might technically exist within the institution [65,66].

Another striking dimension was the palpable sense of helplessness. Several students described scenarios where they recognized the need for better support—either through additional faculty mentorship, more consistent supervision, or simple logistical improvements—but believed that neither the institution nor the local healthcare system could realistically address such demands. One student recalled a pivotal moment in her final clinical rotation as follows:

“I was assigned to a rural health post for my final year clinical placement, and it was severely under-resourced. Each day after my shift, I felt completely drained […] I tried hinting that we needed more direct guidance from our preceptors/clinical facilitators, but they’re also short on time as they are the ones dealing with it […]. It’s like nobody can do much because the problems are so huge—so we just push through.”

(P14, fourth-year student)

In this view, the scarcity of both tangible (e.g., supplies) and intangible (e.g., faculty time) resources appeared to converge, leaving students feeling undervalued and isolated. Crucially, this strain is neither purely psychological nor narrowly contextual: it arises from and is shaped by systemic conditions that extend beyond the reach of personal resilience or small-scale mentoring initiatives [67]. The results mirror certain strands in the global nursing education literature, where chronic underfunding and minimal staffing have been linked to a lowered sense of professional self-efficacy among students [10,68]. However, the accounts here highlight a distinctly moral dimension—participants frame their reluctance to seek help or rest as almost virtuous, given the severe deficits surrounding them.

At the same time, a minority of students took a more critical perspective, suggesting that accepting these shortages at face value might normalize subpar conditions. One third-year student voiced that, “If we never talk about how these resource gaps affect us, nobody will think it’s a priority to fix them.” In her view, silence maintained the status quo, which could erode not only individual wellbeing but also the collective capacity to advocate for systemic reforms. This stance aligns with arguments that attribute high rates of burnout to an education pipeline that systematically underprepares students for effective advocacy in low-resource contexts [10,13]. According to such critiques, while students acquire clinical skills, they often lack the institutional backing or confidence to agitate for change, and the pressure to remain stoic only intensifies this passivity.

Taken together, these narratives articulate a landscape in which urgent and unaddressed deficits in the healthcare system continually overshadow mental health discussions. Students adapt and find personal coping mechanisms—some beneficial, some potentially risky—but their capacity to voice legitimate concerns is constrained by a pervasive sense that systemic crises will always take precedence. This dynamic underscores the potential for a dangerous interplay between self-silencing and institutional complacency, where each side tacitly assumes that mental health improvements cannot or should not rank high on the agenda. While previous scholarship often focuses on how to strengthen students’ coping or resilience skills, the present findings indicate a deeper structural imperative: unless resource inequities and broader system-level issues are tackled, interventions targeting mental wellbeing risk being piecemeal or underutilized.

## 5. Final Considerations

### 5.1. Situated Knowledge and Transferability

The findings should be read as *situated knowledge* produced within a single, large urban tertiary college and its affiliated teaching hospitals. Several features delimit transferability, as follows: (i) the sample was all-female (*n* = 15)—consistent with Nepal’s nursing workforce—so the perspectives of male or gender-diverse students in other contexts are not represented; (ii) participants were aged 23–28 and drawn from second-, third-, and fourth-year cohorts, thereby excluding first-year entrants and mature-age students; (iii) participation was voluntary, introducing the possibility of self-selection toward students motivated to speak about wellbeing; (iv) the site operates an English-medium curriculum within an urban service network, whereas rural placements and non-English-medium programs may configure stressors and supports differently; and (v) clinical teaching at this institution is shared between college-employed faculty (academic oversight, assessment, debriefing) and ward-based preceptors (registered nurses employed by the hospital who supervise day-to-day clinical performance). This division of roles—and the authority gradients it creates—may vary across institutions and countries, limiting straightforward extrapolation.

Rather than claim statistical generalizability, the study offers *analytical transferability*: readers can judge resonance with programs that share key contextual conditions. The four themes—discreet peer help, the performance of resilience, hierarchical constraint, and systemic overshadowing—are likely to be most transferable where authority gradients are pronounced, resource scarcity is persistent, and formal student-support infrastructures are emergent. Conversely, programs characterized by low student-to-preceptor ratios, embedded mental-health services, and flatter pedagogical hierarchies may observe different configurations of stressors and coping.

### 5.2. Recommendations

A holistic approach to promoting mental health in undergraduate nursing students requires balancing individual resilience with a robust support infrastructure. First, the data suggest that while personal coping skills are essential, overly individualizing mental health can overlook the broader organizational and cultural factors at play. Institutions that cultivate open dialogue—in classrooms, simulation labs, or informal peer settings—reduce students’ self-reliance burden and encourage them to voice concerns without fear of reprimand or stigma. Second, transitioning from top-down faculty–student interactions to more collaborative pedagogical methods—such as shared decision making, guided reflection, and consistent debriefs—can mitigate fear of authority and humanize the learning experience. Third, fostering a “family” culture by formally acknowledging and supporting peer-based coping networks allows for mutual care and shared responsibility, countering the sense of isolation that often arises in hierarchical systems with constrained resources. Finally, institutional accountability must be reinforced: improving systems and structural supports—through transparent leadership, adequate staffing, and regular policy evaluations—will ensure that organizational shortcomings do not undermine individual or peer-level efforts.

### 5.3. Directions for Future Research

Several avenues present themselves for extending this research. First, multi-site comparisons within Nepal or across neighboring LMICs could clarify whether the identified patterns—such as reliance on covert peer networks or widespread acceptance of resource scarcities—are institution-specific or regionally pervasive. Second, intervention-based studies might explore faculty development programs that train educators in reflective, student-centered pedagogies, assessing their effect on reported student wellbeing. Finally, a mixed-methods design could complement the depth of RTA with survey-based metrics on anxiety or burnout, providing a more comprehensive understanding of mental health outcomes in these training contexts.

### 5.4. Concluding Reflections

This study presents a context-rich account of how cultural traditions, resource limitations, and hierarchical norms converge to shape undergraduate nursing students’ mental health and wellbeing. Across four thematic strands—ranging from disguised distress to being systemically overwhelmed—students demonstrated adaptive creativity while also revealing vulnerabilities that often remain hidden. These findings have considerable implications for curriculum development, suggesting the integration of structured support mechanisms with theoretical education. Additionally, they highlight the necessity for institutional policies to explicitly address the emotional requirements of future healthcare professionals. Ultimately, recognizing and addressing the interplay of individual coping strategies with entrenched systemic constraints is essential to fostering a nursing workforce that is both clinically proficient and psychologically resilient.

## Figures and Tables

**Table 1:** Sociodemographic characteristics of participants.

Participant	Age	Gender	Year of Study	First-Choice Career?
P1	23	F	2nd Year BSc Nursing	Yes
P2	24	F	2nd Year BSc Nursing	Yes
P3	25	F	2nd Year BSc Nursing	No
P4	23	F	2nd Year BSc Nursing	Yes
P5	24	F	3rd Year BSc Nursing	Yes
P6	25	F	3rd Year BSc Nursing	Yes
P7	26	F	3rd Year BSc Nursing	No
P8	24	F	3rd Year BSc Nursing	No
P9	25	F	3rd Year BSc Nursing	Yes
P10	24	F	4th Year BSc Nursing	Yes
P11	27	F	4th Year BSc Nursing	Yes
P12	26	F	4th Year BSc Nursing	No
P13	25	F	4th Year BSc Nursing	Yes
P14	28	F	4th Year BSc Nursing	No
P15	25	F	4th Year BSc Nursing	Yes

**Table 2:** Semi-structured questions.

No.	Interview Questions
1	Can you describe a typical day in your nursing program, focusing on academic tasks and clinical rotations?
2	Could you share any moments or situations where you felt particularly stressed or anxious during your nursing training?
3	In what ways do you cope with the pressures arising from clinical practice and academic deadlines?
4	Have you observed any differences in how you handle stress now, compared to when you started the BSc Nursing program?
5	How do cultural or familial expectations shape your experiences of coping or seeking support when distressed?
6	How would you describe the role of peers, faculty, or senior nurses in influencing your mental wellbeing?
7	If you maintained reflective notes or diaries, can you discuss how noting down your thoughts affected your coping?
8	What aspects of the nursing curriculum, if any, help you manage stress or learn about mental health?
9	Have you encountered hierarchical issues within your training, and how do they affect your emotional health?
10	Are there changes you believe the institution could make to better address undergraduate nursing students’ wellbeing?

**Table 3:** Illustrative progression from meaning units to emerging themes.

Meaning Unit	Initial Interpretive Segment	Initial Label	Emerging Interpretive Grouping	Emerging Theme
*“Even if I’m overwhelmed, I’d rather not say anything to the instructors. It’s like I’d be seen as not coping.” (P2)*	Reluctance to disclose stress for fear of judgment	Fear of stigma	Culture of silence, reluctance to admit distress	Shrouded Voices, Quiet Connections
*“I only talk to my best friend; we share everything late at night on chat, so it feels safer.” (P5)*	Peer-to-peer emotional support outside formal structures	Quiet peer solidarity	Need for private spaces to share anxieties	Shrouded Voices, Quiet Connections
*“I try to show I’m strong, especially around the doctors. They might think I’m not cut out for nursing if I complain.” (P3)*	Masking genuine struggles to appear competent	Performing fortitude	Personal image management, anxiety about “professional competence”	Performing Resilience: Masking Authentic Struggles
*“No one really checks if we’re okay. They assume we can handle it, so I just keep smiling—like I’m supposed to be the perfect nurse.” (P14)*	Internalization of idealized nursing persona	Suppression of distress	Societal and institutional expectations lead to emotional concealment	Performing Resilience: Masking Authentic Struggles
*“When senior staff say, ‘You must do it this way,’ I don’t argue—even if I’m unsure it’s right. It’s how we’ve been taught.” (P7)*	Acceptance of top-down directives without question	Unquestioning compliance	Cultural and institutional hierarchy shaping student deference	Power, Hierarchy, and the Weight of Tradition
*“I wanted to speak up about the workload, but the head nurse said, ‘Know your place.’ So I stayed quiet.” (P13)*	Discouragement from voicing concerns	Silencing under authority	Reinforcement of vertical power structures	Power, Hierarchy, and the Weight of Tradition
*“Sometimes we’re out of basic supplies, and families can’t afford them. I feel guilty complaining about my stress when they have it worse.” (P1)*	Comparing personal distress to systemic patient burdens	Guilt in face of larger crises	Deprioritizing self-care due to resource deficiencies and patient hardships	Overshadowed by Systemic Realities: Resource Scarcity and Larger Crises
*“I see the hospital short-staffed, so I think, ‘At least I can help a little.’ But it’s tough: I’m exhausted, yet feel wrong about focusing on my own well-being.” (P6)*	Moral tension between self-care and meeting institutional gaps	Self-sacrifice	Systemic under-resourcing intersects with personal wellbeing	Overshadowed by Systemic Realities: Resource Scarcity and Larger Crises

## Data Availability

The original contributions presented in this study are included in the article. Further inquiries can be directed to the corresponding author.

## Supplementary Materials

The following supporting information can be downloaded at: https://www.mdpi.com/article/10.3390/nursrep15090312/s1, Table S1: Progression from Meaning Units to Themes via Reflexive Interpretation (illustrative segment).

## Public Involvement Statement

There was no public involvement in any aspect of this research.

## Guidelines and Standards Statement

This study follows the Reflexive Thematic Analysis Reporting Guidelines (RTARG) guidelines for qualitative studies.
