# Supplementary material for: Concealing, Connecting, and Confronting: A Reflexive Inquiry into Mental Health and Wellbeing Among Undergraduate Nursing Students
Source: Nurs Rep. 2025 Aug 25;15(9):312. doi: 10.3390/nursrep15090312 (PMC12472999; doi:10.3390/nursrep15090312)
Supplement: Supplementary file 1 [file nursrep-15-00312-s001.zip › nursrep-3799766-supplementary.pdf]

Supplementary File. Reflexive Decision Log (Representative Segment)

Table S1. Progression from Meaning Units to Themes via Reflexive Interpretation (illustrative segment)

**Meaning Unit (participant, year)**	**Initial Interpretive Segment**	**Initial Label (provisional)**	**Reflexive memo (excerpt)**	**Emerging Interpretive Grouping**	**Emerging Theme**
"Even if I'm overwhelmed, I'd rather not say anything to the instructors. It's like I'd be seen as not coping." (P2, 2nd)	Reluctance to disclose distress under evaluative gaze	Self-silencing to protect competence	I initially read this as "silence = weakness," but memoing reminds me it may be a safety strategy in a high-stakes, graded space. Resist moralizing the silence; attend to context.	Stigma and concealment; managing impressions; evaluative scrutiny	Shrouded Voices, Quiet Connections
"I only talk to my best friend; we share everything late at night on chat, so it feels safer." (P5, 3rd)	Seeking discreet, relational sanctuary	Quiet peer solidarity	My educator lens wants to 'formalize' this support; memo cautions against imposing institutional frames—students curate micro-shelters that feel trustworthy.	Private peer havens; relational buffering	Shrouded Voices, Quiet Connections
"I try to show I'm strong, especially around the doctors. They might think I'm not cut out for nursing if I complain." (P3, 2nd)	Managing a competent persona under hierarchy	Performing fortitude	I feel the pull to call this "coping," yet the talk of audience and appearance points to performance. Keep the focus on the masking dynamic, not individual blame.	Impression management; idealized professional self	Performing Resilience: Masking Authentic Struggles

**Meaning Unit (participant, year)**	**Initial Interpretive Segment**	**Initial Label (provisional)**	**Reflexive memo (excerpt)**	**Emerging Interpretive Grouping**	**Emerging Theme**
"No one really checks if we're okay… I just keep smiling—like I'm supposed to be the perfect nurse." (P14, 4th)	Internalizing the "perfect nurse" ideal	Suppression of distress	Memo asks: Am I over-reading "shame"? Keep interpretive distance—note how some students describe pressure to perform calmness.	Institutional/ social expectations; emotional concealment	Performing Resilience: Masking Authentic Struggles
"When senior staff say, 'You must do it this way,' I don't argue—even if I'm unsure it's right." (P7, 3rd)	Deference to authority; non-questioning compliance	Hierarchical acquiescence	My assumption that all hierarchy is harmful is simplistic; memo: differentiate mentorship from silencing—here, the student signals uncertainty + deference.	Top-down directives; constrained voice	Power, Hierarchy, and the Weight of Tradition
"I wanted to speak up about the workload, but the head nurse said, 'Know your place.' So I stayed quiet." (P13, 4th)	Direct discouragement of voice	Silencing under authority	Memo: Strong directive language. Attend to the effect (quieting) without generalizing to all wards. Mark as an example of boundary-policing.	Boundary-policing; sanctioned silence	Power, Hierarchy, and the Weight of Tradition
"Sometimes we're out of basic supplies, and families can't afford them. I feel guilty complaining about my	Moral comparison with patients' hardship	Guilt-mediated self-erasure	I gravitate to "altruism," but memo warns: this can be a costly moral calculus—students	Resource scarcity; moral incongruence	Overshadowed by Systemic Realities: Resource Scarcity and Larger Crises

Meaning Unit (participant, year)	Initial Interpretive Segment	Initial Label (provisional)	Reflexive memo (excerpt)	Emerging Interpretive Grouping	Emerging Theme
stress when they have it worse." (P1, 2nd)			deprioritize self-care amid structural scarcity.		
"I see the hospital short-staffed… I'm exhausted, yet feel wrong about focusing on my own well-being." (P6, 3rd)	Self-sacrifice in response to systemic gaps	Normalizing over-extension	Memo flags risk of romanticizing sacrifice; hold onto the system–self tension (under-resourcing ↔ deferred self-care).	Systemic under-resourcing; deferred self-care	Overshadowed by Systemic Realities: Resource Scarcity and Larger Crises

Notes: (i) P-codes are pseudonymous; year refers to academic year.

(ii) "Initial labels" were working sense-markers, not fixed "codes"; their purpose was to keep interpretive possibilities open while moving toward theme construction consonant with RTA's non-positivist stance.

(iii) This table is illustrative rather than exhaustive; additional entries are available on request as part of the reflexive decision trail.
